# Effectiveness of Telemedicine vs Face-to-Face Consultation in Fighting COVID-19: Retrospective Cohort Study of Adult Patients With COVID-19 in a Primary Care Setting

**DOI:** 10.2196/74046

**Published:** 2026-05-14

**Authors:** Fangfang Jiao, Ka Ming Ho, Lapkin Chiang, Siu Hin Ko, Catherine Xiaorui Chen

**Affiliations:** 1 Department of Family Medicine and Primary Health Care Queen Elizabeth Hospital Hong Kong SAR China (Hong Kong)

**Keywords:** telemedicine, designated clinic, COVID-19, health service use, tele-designated clinic, physical designated clinic

## Abstract

**Background:**

Telemedicine use expanded rapidly during the COVID-19 pandemic. The Hong Kong Hospital Authority (HA) launched both tele-designated clinics (Tele-DCs) and face-to-face physical designated clinics (PDCs) to manage mild cases. However, the comparative effectiveness of these models remains unclear.

**Objective:**

This study aimed to compare clinical outcomes, specifically hospitalization and severe complications, between patients with mild COVID-19 managed via Tele-DCs versus PDCs in Hong Kong’s public primary care setting.

**Methods:**

We conducted a retrospective cohort study involving all patients with COVID-19, aged 18 years or older, who visited a PDC (n=23,031) or a Tele-DC (n=38,628) at the Kowloon Central Cluster in Hong Kong from July 28, 2022, to January 29, 2023. Patients were matched 1:1 using propensity score matching based on age, sex, smoking status, comprehensive social security assistance (CSSA) status, and the Charlson comorbidity score, resulting in 17,199 patients per group. The primary outcome was the hospital admission rate between day 1 and day 28. Secondary outcomes included severe complications, mortality, accident and emergency department (AED) use, the antiviral prescription rate, and DC revisit.

**Results:**

The average age of patients in the Tele-DC and PDC groups was 58.55 (SD 17.53) and 58.53 (SD 17.54) years, respectively (*P*=.93). In both groups, 9.05% (n=1557) of patients were on CSSA, and 11% (n=1892) were smokers. Compared to the PDC group, the Tele-DC group demonstrated similar hospital admission rates (Tele-DC: n=497, 2.89%; PDC: n=471, 2.74%; between-group difference 0.15%, 95% CI –0.20% to 0.50%, *P*=.40), lengths of stay (Tele-DC: mean 6.92, SD 0.47 days; PDC: mean 6.61, SD 0.50 days; between-group difference 0.31 days, 95% CI –1.65 to 1.04, *P*=.66), severe complication rates (Tele-DC: n=46, 0.27%; PDC: n=33, 0.19%; between-group difference 0.08%, 95% CI –0.03% to 0.18%, *P*=.18), and mortality rates (Tele-DC: n=23, 0.13%; PDC: n=18, 0.10%; between-group difference 0.03%, 95% CI –0.04% to 0.10%, *P*=.39). However, the Tele-DC group exhibited a higher AED visit rate (n=641, 3.73%, vs n=542, 3.15%; between-group difference 0.58%, 95% CI 0.19%-0.96%, *P*.003) and DC revisit rate (n=1446, 8.41%, vs n=1287, 7.48%; between-group difference 0.93%, 95% CI 0.09%-1.50%, *P*.002). In addition, the Tele-DC group had a lower antiviral prescription rate (n=9872, 57.4%, vs n=10,797, 62.78%; between-group difference –5.38%, 95% CI –6.41% to –4.32%, *P*<.001).

**Conclusions:**

The tele-DC demonstrated clinical safety comparable to the PDC regarding hospitalization and severe complications for patients with mild COVID-19. By validating a scalable model without complex home monitoring, these findings challenge the strict necessity of physical examinations for safe triage and support a digital-first strategy for future infectious surges. However, the disparities observed in AED visits and antiviral prescription rates suggest that integrated remote monitoring tools and improved medication logistics are needed to fully replicate the efficacy of conventional care.

## Introduction

COVID-19 was a global pandemic that had a devastating impact on health care worldwide. COVID-19 is highly contagious, with more than 762 million identified cases and 6.8 million deaths reported worldwide as of April 2023 [1]. Although severe and critical complications predominantly occur in older adults or those with certain underlying medical comorbidities [2], the sheer volume of mild-to-moderate cases has historically overwhelmed health care systems, necessitating rapid adaptations in service delivery models to maintain continuity of care, while minimizing transmission risks [3].

In January 2022, Hong Kong experienced the fifth wave of the COVID-19 pandemic. This wave proved to be significantly more severe than its predecessors, with thousands of daily infections placing unprecedented strain on existing health care facilities. To meet the increasing service demand from patients with confirmed COVID-19 in the community, the Hong Kong Hospital Authority (HA) converted several general outpatient clinics (GOPCs) into COVID-19 designated clinics (DCs) starting in January 2022 [4]. Furthermore, to curb the spread of COVID-19 and improve health care accessibility, the HA introduced telehealth services in the form of tele-designated clinics (Tele-DCs) on July 28, 2022. The objective of both physical designated clinics (PDCs) and Tele-DCs was to provide prompt treatment for patients with mild COVID-19 and in a stable clinical condition. Both PDCs and Tele-DCs operated until January 29, 2023, when the Hong Kong government lifted the isolation order for patients with COVID-19 [5].

Telemedicine has several well-established advantages over traditional face-to-face consultation for managing infectious diseases. First, remote consultation via teledevices reduces the risk of physical transmission of communicable illnesses. Second, it facilitates clinical triage, avoiding overwhelming emergency and in-patient services [6]. Third, it improves access to medical care, particularly for patients with mobility issues. Consequently, teleconsultation rapidly gained popularity following the pandemic’s onset. It has been effectively used to manage a range of conditions, including diabetes mellitus [7], cancer [8], acute pediatric conditions [9], orthopedic conditions, psychological diseases, cardiovascular diseases, and chronic pain [10].

However, the rapid implementation of telemedicine has outpaced evidence regarding its comparative safety for acute respiratory infections. A critical limitation of teleconsultation is the inability to perform physical examinations, such as lung auscultation, or to obtain immediate vital signs, which are traditional cornerstones of pneumonia assessment [11,12]. Although remote patient monitoring using home oximetry has shown promise in mitigating these risks, such programs are resource intensive and difficult to scale during massive surges [13,14].

The shift to telemedicine in primary care during the COVID-19 pandemic has been extensively documented [15,16], yet studies evaluating its effectiveness in COVID-19–specific care have yielded mixed results. A large cohort study in Spain found that the proactive telemonitoring and teleconsultation for patients with COVID-19 are associated with fewer emergency department (ED) visits, lower hospitalization rates, a shorter length of stay (LOS), and lower mortality when compared with other models of care [17]. Conversely, a cohort study in the United States for post–ED visit follow-up care patients with COVID-19 found that telehealth is associated with increased rates of ED visits and hospitalizations compared with in-person care [18]. Although some studies have indicated high patient satisfaction with telemedicine for various conditions, including COVID-19 [19], robust comparative effectiveness studies, particularly cluster-based cohort studies that account for the safety of consultation-only telemedicine, remain scarce.

This knowledge gap is critical: health care systems need to know whether a scalable, consultation-based telemedicine model (Tele-DC) is as safe as a face-to-face model involving physical examination (PDC) for triaging mild COVID-19 cases. To address this gap, this study aimed to compare the risk of hospitalization and severe complications among patients with mild COVID-19 managed via Tele-DCs versus those managed via PDCs in a public primary care setting in Hong Kong. We hypothesized that Tele-DCs are associated with hospitalization and severe complications rates compared to PDCs. We believe that the findings from this study will provide essential evidence for future service planning, specifically regarding the safety of substituting physical examinations with virtual assessments during infectious surges.

## Methods

### Study Design

We conducted a retrospective cohort study to compare clinical outcomes between Tele-DCs and PDCs in the management of patients with COVID-19. Patients in both groups were matched using propensity score matching based on age, sex, smoking status, comprehensive social security assistance (CSSA) status, and the Charlson comorbidity score [20]. This study adhered to the STROBE (Strengthening the Reporting of Observational Studies in Epidemiology) guidelines [21].

Propensity score matching pairs subjects based on observable characteristics that indicate a similar probability of receiving treatment (similar propensity score), even though they receive different interventions. The propensity score is independent of the outcomes and represents the conditional probability of receiving an intervention, given the observed baseline characteristics. Propensity score matching is suitable for studies with a large sample size and many covariates as it helps balance the distribution of covariates between treatment groups [22]. We used propensity score matching to reduce confounding at baseline.

The Charlson comorbidity index is a weighted index used to predict the risk of death within 1 year of hospitalization for patients with specific comorbid conditions. This index includes 17 conditions, and each condition is assigned a weight ranging from 1 to 6. These weights are determined based on the estimated 1-year mortality hazard ratio derived from a Cox proportional hazards model. These weights were summed up to produce the Charlson comorbidity score. The conditions incorporated into the Charlson comorbidity score encompassed a wide range of comorbidities identified as risk factors for severe COVID-19. These included cancer, chronic lung disease, stroke, chronic kidney disease (CKD), chronic liver disease, diabetes mellitus, heart conditions, HIV, and more [23].

### Study Setting

Since July 28, 2022, the HA has launched DC services in telehealth mode (Tele-DCs) for the management of confirmed cases of COVID-19 with mild symptoms in the community. Individuals aged 18 years and above who had received a positive diagnosis for COVID-19 infection through either a rapid antigen test (RAT) or a COVID-19 polymerase chain reaction (PCR) test could book appointments for the Tele-DC service using the government-developed mobile app HA GO. This app features encrypted real-time video capabilities for teleconsultations, allowing Tele-DC doctors to conduct consultations on an iPad. During the consultation, doctors assessed patients’ condition, provided medical treatment, and offered self-care advice to suitable patients via the electronic device. Unstable patients or those at high risk of developing critical conditions were advised to visit the accident and emergency department (AED), as determined by the doctors. For consultations conducted during the morning session, prescribed medications were delivered to patients’ registered addresses by the afternoon on the same day. For consultations conducted during the afternoon session, medications were delivered to patients on the following morning. In addition, patients in need also received a pulse oximeter, along with the medications.

The PDC was established in January 2022 and provided face-to-face consultations to patients with mild COVID-19. Prior to consultations, nurses checked every patient’s temperature and oxygen saturation using a pulse oximeter. SSurgery tests were available in the PDC, including electrocardiography (ECG) , blood glucose monitoring using Glucometer (Contour Plus Elite, PHC Corporation), and Urine Multistix test. The doctors assessed the patients’ condition, provided treatment, and advised unstable patients to attend the AED in the same manner as in the Tele-DC. Following the consultation, medications were dispensed to the patients right away.

Both the Tele-DC and the PDC were free of charge to patients. The attending doctors were the same as the GOPC doctors and had a similar working experience. The assigned consultation time for the Tele-DC and the PDC was similar. As a small proportion of patients had multiple visits at the Tele-DC or the PDC, patient groups were determined based on their first visit to either DC. The Tele-DC and PDC groups were defined as the intervention and control groups, respectively.

### Participants

The inclusion criteria were that all patients with COVID-19 had visited one HA Kowloon Central Cluster PDC or Tele-DC from July 28, 2022, to January 29, 2023. During this period, both DC modes were available in the HA for patients to choose from. A COVID-19 diagnosis was identified using International Classification of Primary Care-2 (ICPC-2) A77 code, which includes a diagnosis of COVID-19; Coxsackie virus infection; dengue fever; hand, foot, and mouth disease; and adenovirus infection. When patients booked a PDC appointment over the phone or a Tele-DC appointment through the mobile app, they were reminded that only patients with confirmed COVID-19 were eligible for the booking. Therefore, patients with other illnesses under code A77 were excluded at the time of booking. Furthermore, when nurses initially assessed patients in the PDC or the Tele-DC, they asked and documented the diagnostic method for COVID-19 (either PCR or RAT).

The exclusion criteria were patients younger than 18 years old or patients without a diagnosis code of A77.

### Assessments

The primary outcome was the hospital admission rate from day 1 to day 28 after the first visit to the Tele-DC or the PDC. The secondary outcomes included AED visits between day 1 and day 28, the hospital admission rate and AED visit on the same day (day 0) after Tele-DC or PDC visit, the LOS, intensive care unit (ICU) or high-dependency unit (HDU) admission, the revisit rate of the Tele-DC or the PDC after the first consultation at the Tele-DC or the PDC, prescriptions of antivirals, mortality, and development of COVID-19–related severe and critical complications (including pneumonia, sepsis, encephalopathy/encephalitis, myocarditis, systemic inflammatory response syndrome, and shock) within 28 days after the first Tele-DC or PDC visit. To cancel out the effect of antivirals on the severity of COVID-19, we further conducted a subgroup analysis of health service use by prescription of antivirals.

### Measurements

Data were collected from the Clinical Management System (CMS) or retrieved from the Clinical Data Analysis and Reporting System (CDARS) of the HA and included the following:

- Baseline characteristics of patients, including sex, age, smoking status, CSSA status, and existing comorbidities. The CSSA is a welfare scheme that provides supplementary payments to Hong Kong residents whose income is not sufficient to meet basic needs. It is used as an indicator of socioeconomic status. The comorbidities at baseline were identified using the *International Classification of Disease, Ninth Revision, Clinical Modification* (ICD-9-CM) codes and ICPC-2 codes (Table S1 in Multimedia Appendix 1).
- Prescription of antivirals (ie, Paxlovid or molnupiravir, two choices available at both the Tele-DC and the PDC).
- Health service use rate on day 0 of visit to the Tele-DC or the PDC, which included AED visits and hospital admission.
- Health service use rates between day 1 and day 28 after the first consultation at the Tele-DC or the PDC, including AED visits, hospital admission, the LOS, and ICU/HDU admission.
- The revisit rate of the Tele-DC or the PDC within 28 days after the first consultation at the Tele-DC or the PDC.
- The development of severe complications within 28 days after COVID-19 diagnosis. These complications, including pneumonia, sepsis, encephalopathy/encephalitis, myocarditis, systemic inflammatory response syndrome, and shock, were identified using ICD-9-CM codes (Table S2 in Multimedia Appendix 1).
- Mortality within 28 days after the first visit to at the Tele-DC or the PDC.

As patients with COVID-19 were managed only in the public health sector in Hong Kong, and the clinical data of the public health sector were all available in the CMS and the CDARS, there were no missing data or loss of data at follow-up in this study. All patients were followed up for 28 days.

### Sample Size

We estimated that 3% patients with COVID-19 would require inpatient care during the fifth wave in Hong Kong [24]. We assumed a relative risk of 1.3 between the two groups as clinical significance, with a noninferior limit of 0.9%. We used 80% power (1 – β=80%) and a 95% CI (α .05). Using a sample size calculator [25] for noninferiority studies, 6156 individuals would be needed in each group. To allow for a 20% exclusion rate, a minimum of 7388 individuals would be needed in each group for data analysis. To reduce selection bias, we included all eligible patients in the data analysis.

### Data Analysis

For descriptive statistics, continuous variables were expressed as mean (SD) and categorical variables were expressed as percentages. We used the independent *t* test or chi-squared test, as appropriate, to compare demographics, clinical parameters, and health service use between groups. To assess noninferiority for the primary outcome of hospital admission rate, we calculated the absolute risk difference between the Tele-DC and PDC groups, along with its 95% CI. Noninferiority was considered established if the upper bound of the 95% CI for the risk difference was less than the predefined noninferiority margin of 0.9%. The *psmatch2* STATA package was used to conduct propensity score matching. We estimated the average treatment effect on the treated (ATT). We adopted a one-to-one matching without replacement with a caliper of 0.0001, which means the differences in propensity scores for each matched pair were no greater than 0.0001. Unmatched individuals were discarded. No sensitivity analysis was performed in this study. All statistical analyses were performed using STATA/MP (StataCorp), and *P*<.05 was considered statistically significant.

### Ethical Considerations

This study was approved by the Research Ethics Committee (Kowloon Central/Kowloon East; reference #KC/KE-23-0092/ER-3). Informed consent was waived by the ethics committee because this retrospective observational study involved only the analysis of deidentified data collected for routine clinical care, with no direct patient contact. All the study data were deidentified. No compensation was provided to participants, as there was no direct contact with patients and no active participation required. No images or supplementary materials in this manuscript contain identifiable information about individual participants. No patient photographs or personal health information are included.

## Results

### Basic Patient Characteristics

**Figure 1:**
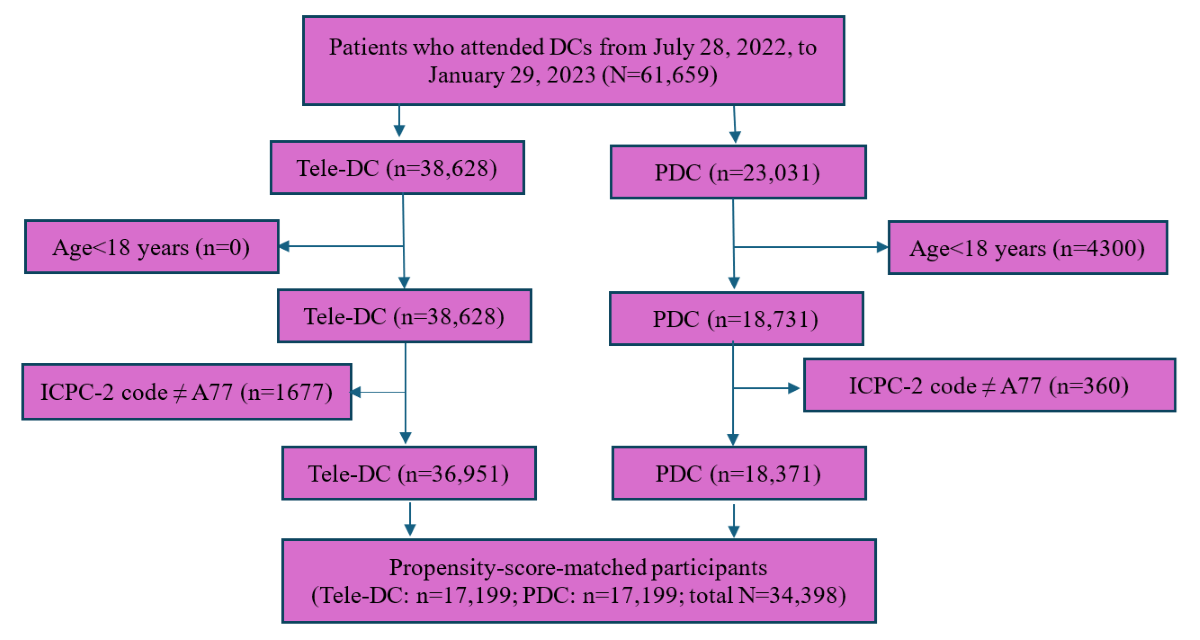
Flowchart of participant selection. DC: designated clinic; ICPC-2: International Classification of Primary Care-2; PDC: physical designated clinic; Tele-DC: tele-designated clinic.

A total of 61,659 patients visited either the Tele-DC or the PDC during the study period. Among them, 23,031 (37.35%) visited the PDC, of which 4300 (18.67%) were younger than 18 years old. After excluding patients without a diagnosis of COVID-19, there were 36,951 (59.93%) patients in the Tele-DC group and 18,371 (79.77%) patients in the PDC group (Figure 1).

Before propensity score matching, patients in the Tele-DC group were significantly younger than those in the PDC group (mean 55.30, SD 17.63, years vs mean 59.31, SD 17.57, years; *P*<.001). The proportion of patients on CSSA was significantly higher in the PDC group (n=2039, 11.1%) than in the Tele-DC group (n=2465, 6.67%; *P*<.001). The PDC group also showed baseline comorbidities, as indicated by the Charlson comorbidity index (mean 0.10, SD 0.42) compared to the Tele-DC group (mean 0.04, SD 0.28; *P*<.001), as shown in Table 1.

**Table 1:** Basic characteristics of study participants before propensity score matching.

Characteristics		Tele-DC^a^ (n=36,951)	PDC^b^ (n=18,371)	Standardized mean difference	*P* value
Age (years), mean (SD)		55.30 (17.63)	59.31 (17.57)	–22.80	<.001^c^
**Age group (years), n (%)**					
	18-29	2727 (7.38)	1034 (5.62)	—^d^	<.001^c^
	30-44	8853 (23.96)	3331 (18.13)	—	—
	45-59	8477 (22.94)	3794 (20.65)	—	—
	60-74	11,806 (31.95)	6617 (36.02)	—	—
	≥75	5088 (13.77)	3599 (19.59)	—	—
Female, n (%)		22,766 (61.61)	10,819 (58.89)	5.60	<.001^c^
Current or ex-smokers, n (%)		3769 (10.20)	2379 (12.95)	9.00	<.001^c^
Patients on CSSA^e^, n (%)		2465 (6.67)	2039 (11.10)	–15.60	<.001^c^
Charlson comorbidity index, mean (SD)		0.04 (0.28)	0.10 (0.42)	–17.50	<.001^c^

^a^Tele-DC: tele-designated clinic.

^b^PDC: physical designated clinic.

^c^Not applicable.

^d^Significant *P* values.

^e^CSSA: comprehensive social security assistance.

After propensity score matching, each group had 17,199 patients. The average age in both groups was 58.55 (SD 17.53) years. Females accounted for almost the same proportion in both groups: 59.53% (n=10,239) of patients in the Tele-DC group and 59.50% (n=10,233) of patients in the PDC group. Similarly, the proportion of patients on CSSA was 9.05% (n=1557) in both groups. The Charlson comorbidity index was also similar in each group (Tele-DC: mean 0.05, SD 0.28; PDS: mean 0.04, SD 0.27; *P*=.77), as shown in Table 2.

**Table 2:** Basic characteristics of study participants after propensity score matching.

Characteristics		Tele-DC^a^ (n=17,199)		PDC^b^ (n=17,199)		Standardized mean difference		*P* value
Age (years), mean (SD)		58.55 (17.53)		58.53 (17.54)		–0.001		.93
**Age group (years), n (%)**								
	18-29	1013 (5.89)	1018 (5.92)		—^c^		.99	
	30-44	3287 (19.11)	3287 (19.11)		—		—	
	45-59	3658 (21.27)	3658 (21.27)		—		—	
	60-74	6094 (35.43)	6094 (35.43)		—		—	
	≥75	3147 (18.30)	3142 (18.27)		—		—	
Female, n (%)		10,239 (59.53)		10,233 (59.50)		0.001		.96
Current or ex-smokers, n (%)		1894 (11.01)		1892 (11.00)		0.002		.99
Patients on CSSA^d^, n (%)		1557 (9.05)		1557 (9.05)		0.000		.99
Charlson comorbidity index, mean (SD)		0.05 (0.28)		0.04 (0.27)		0.002		.77

^a^Tele-DC: tele-designated clinic.

^b^PDC: physical designated clinic.

^c^Not applicable.

^d^CSSA: comprehensive social security assistance.

### Health Service Use

The primary outcome was the hospital admission rate from day 1 to day 28 after visiting the DCs, which was similar between the Tele-DC (2.89%) and and PDC (2.74%) groups, respectively (*P*=.40). The absolute between-group difference was 0.15% (95% CI –0.20% to 0.50%). Because the upper bound of the 95% CI (0.50%) was well below the predefined noninferiority margin of 0.9%, the Tele-DC was deemed noninferior to the PDC for the primary outcome. The average LOS was comparable between the two groups (Tele-DC: mean 6.92, SD 0.47 days; PDC: mean 6.61, SD 0.50 days; *P*=.66). In both groups, 15 (0.09%) patients required ICU or HDU admission. The proportion of patients admitted to the hospital on day 0 was similar (Tele-DC: n=108, 0.63%; PDC: n=86, 0.50%; *P*=.11). However, more patients in the Tele-DC group (n=641, 3.73%) visited the AED within 1-28 days than in the PDC group (n=542, 3.15%; *P*=.003). The proportion of patients visiting the AED on day 0 was similar (Tele-DC: n=183, 1.06%; PDC: n=175, 1.02%; *P*=.67), as shown in Table 3.

**Table 3:** Comparison of health service use.

Health care service use			Tele-DC^a^ (n=17,199)		PDC^b^ (n=17,199)		Between-group difference^c^			*P* value^d^
			Value		Value		Percentage (95% CI)	Mean (95% CI)		
**Hospital admission within 1-28 days after DC^e^ visit**										
	Overall admission rate, n (%)	497 (2.89)		471 (2.74)		0.15 (–0.20 to 0.50)		—^f^	.40	
	LOS^g^, mean (SD)	6.92 (0.47)		6.61 (0.50)		—		0.31 (–1.65 to 1.04)	.66	
	HDU^h^/ICU^i^ admission rate, n (%)	15 (0.09)		15 (0.09)		0		—	.99	
	LOS in HDU/ICU, mean (SD)	3.07 (0.70)		7.33 (3.75)		—		–4.27 (–3.55 to 12.08)	.27	
**Hospital admission on day 0 after DC visit**										
	Overall admission rate, n (%)	108 (0.63)		86 (0.50)		0.13 (–0.03 to 0.29)		—	.11	
	LOS, mean (SD)	13.76 (1.57)		10.55 (1.21)		—		3.21 (–7.30 to 0.87)	.12	
	HDU/ICU admission rate, n (%)	5 (0.03)		1 (0.01)		—		—	—	
	LOS in HDU/ICU, mean (SD)	9.00 (6.76)		3 (—)		—		—	—	
**AED^j^ visit, n (%)**										
	Within 1-28 days after DC visit	641 (3.73)		542 (3.15)		0.58 (0.19 to 0.96)		—	.003^k^	
	On day 0 after DC visit	183 (1.06)		175 (1.02)		0.05 (–0.17 to 0.26)		—	.67	
**Proportion of patients with repeated DC visit, n (%)**										
	Patients with second DC visit	1446 (8.41)		1,287 (7.48)		0.93 (0.09 to 1.50)		—	.002^k^	
	Tele-DC visit	1220 (7.09)		373 (2.17)		—		—	<.001^k^	
	PDC visit	226 (1.31)		914 (5.31)		—		—	—	
	Patients with third DC visit	150 (0.87)		129 (0.75)		0.12 (–0.07 to 0.31)		—	.21^k^	
	Tele-DC visit	118 (0.69)		55 (0.32)		—		—	<.001^k^	
	PDC visit	32 (0.19)		74 (0.43)		—		—	—	

^a^Tele-DC: tele-designated clinic.

^b^PDC: physical designated clinic.

^c^Between-group difference = Tele-DC group mean or percentage – PDC group mean or percentage.

^d^*P* values calculated with the independent *t* test or the chi-squared test, as appropriate.

^e^DC: designated clinic.

^f^Not applicable.

^g^LOS: length of stay.

^h^HDU: high-dependency unit.

^i^ICU: intensive care unit.

^j^AED: accident and emergency department.

^k^Significant *P* values.

After visiting the PDC, 7.48% (n=1287) of patients made a second visit to the DC; the majority of these (n=914, 71.01%) chose to revisit the PDC. In contrast, in the Tele-DC group, the revisit rates were slightly higher, with 8.41% (n=1446) of patients visiting the DC again; the majority of these (n=1220, 84.37%) opted to visit the Tele-DC for their second visit (Table 3).

### Prescription of Antivirals

The PDC group exhibited a higher prescription rate for antivirals (n=10,797, 62.78%) than the Tele-DC group (n=9872, 57.40%; *P*<.001). Paxlovid accounted for ~75% of the prescriptions in both groups. Furthermore, the PDC group also had a higher proportion of patients aged 60 years or older who received antivirals (n=7714, 83.47%) compared to the Tele-DC group (n=7147, 77.37%; *P*<.001), as shown in Table 4.

**Table 4:** Prescription of antivirals.

Patient group and antivirals			Tele-DC^a^, n (%)		PDC^b^, n (%)		Between-group difference^c^, percentage (95% CI)		*P* value^d^
**All patients (n=17,199 in each group)**									
	Any antivirals	9872 (57.40)		10,797 (62.78)		–5.38 (–6.41 to –4.34)		<.001	
	Paxlovid	7561 (43.96)		8085 (47.01)		–3.05 (–3.04 to –2.00)		<.001	
	Monulpiravir	2311 (13.44)		2712 (15.77)		–2.33 (–3.08 to –1.59)		<.001	
**Patients aged ≥60 years (Tele-DC: n=9237; PDS: n=9242)**									
	Any antivirals	7147 (77.37)		7714 (83.47)		–6.10 (–7.23 to –4.95)		<.001	
	Paxlovid	5196 (56.25)		5411 (58.55)		–2.30 (–3.72 to –0.87)		.002	
	Monulpiravir	1951 (21.12)		2303 (24.92)		–3.8 (–5.01 to –2.59)		<.001	

^a^Tele-DC: tele-designated clinic.

^b^PDC: physical designated clinic.

^c^Between-group difference = Tele-DC group percentage – PDC group percentage.

^d^*P* values calculated with the chi-squared test. All *P* values were significant.

### Clinical Outcomes of Patients With COVID-19

The proportion of patients who developed severe complications was similar between the Tele-DC and PDC groups. Among patients who visited the AED with or without admission within 1-28 days after visiting the DC, 46 (0.27%) patients in the Tele-DC group and 33 (0.19%) patients in the PDC group developed severe complications (*P*=.18). In addition, 25 (0.15%) patients the Tele-DC group and 17 (0.10%) patients in the PDC group visited the AED on the same day after their DC visit and developed severe complications (*P*=.28). The mortality rate was also similar between the two groups among patients who visited the AED within 1-28 days after visiting the DC: 23 (0.13%) in the Tele-DC group and 18 (0.10%) in the PDC group (*P*=.39), as shown in Table 5.

**Table 5:** Clinical outcomes.

Patient group and AED^a^ visits			Tele-DC^b^ (n=17,199), n (%)		PDC^c^ (n=17,199), n (%)		Between-group difference^d^, percentage (95% CI)		*P* value^e^
**Development of severe complications^f^**									
	AED visit within 1-28 days after DC^g^ visit	46 (0.27)		33 (0.19)		0.08 (–0.03 to 0.18)		.18	
	AED visit on day 0 after DC visit	25 (0.15)		17 (0.10)		0.05 (–0.03 to 0.12)		.28	
**Death within 28 days after** **visit**									
	AED visit within 1-28 days after DC visit	23 (0.13)		18 (0.10)		0.03 (–0.04 to 0.10)		.39	
	AED visit on day 0 after DC visit	10 (0.06)		6 (0.03)		0.03 (–0.02 to 0.07)		.45	

^a^AED: accident and emergency department.

^b^Tele-DC: tele-designated clinic.

^c^PDC: physical designated clinic.

^d^Between-group difference = Tele-DC group percentage – PDC group percentage.

^e^*P* values calculated with the chi-squared test.

^f^Severe complications included pneumonia, sepsis, encephalopathy/encephalitis, myocarditis, systemic inflammatory response syndrome, and shock.

^g^DC: designated clinic.

### Subgroup Analysis of Health Service Use by Prescription of Antivirals

To account for the effects of antiviral use on outcomes, we conducted a subgroup analysis stratified by antiviral prescription. Among patients with an antiviral prescription, the Tele-DC and PDC groups had similar admission rates within 1-28 days after DC visits (n=357, 3.62%, vs n=370, 3.43%; *P*=.46) and a similar LOS (mean 6.42, SD 0.50, vs mean 6.29, SD 0.54; *P*=.87). Among patients without an antiviral prescription, the Tele-DC and PDC groups had comparable admission rates within 1-28 days after DC visits as well (n=140, 1.91%, vs n=101, 1.58%; *P*=.14). The hospital admission rate on day 0 after DC visits was also similar between the Tele-DC and PDC groups, regardless of whether antivirals were prescribed. Of note, among patients without an antiviral prescription, the Tele-DC group had more AED visits within 1-28 days (n=252, 3.44%) compared to the PDC group (n=152, 2.37%; *P*<.001), while among patients with an antiviral prescription, the Tele-DC group had a higher proportion of patients with a second DC visit (n=906, 9.18%) compared to the PDC group (n=856, 7.93%; *P*<.001), as shown in Table 6.

**Table 6:** Comparison of health service use by antiviral prescription subgroups.

Health care service use			Tele-DC^a^ (n=17,199)		PDC^b^ (n=17,199)		Between-group difference^c^				*P* value^d^
			Value		Value		Percentage (95% CI)		Mean (95% CI)		
**Patients with antiviral prescription and hospital admission within 1-28 days after DC^e^ visit (Tele-DC: n=9872; PDC: n=10,797)**											
	Overall admission rate, n (%)	357 (3.62)		370 (3.43)		0.19 (–0.31 to 0.69)		—^f^		.46	
	LOS^g^, mean (SE)	6.42 (0.50)		6.29 (0.54)		—		0.13 (–1.34 to 1.59)		.87	
**Patients without antiviral prescriptionand hospital admission within 1-28 days after DC visit (Tele-DC: n=7327; PDC: n=6402)**											
	Overall admission rate, n (%)	140 (1.91)		101 (1.58)		0.33 (–0.10 to 0.77)		—		.14	
	LOS, mean (SE)	7.86 (0.95)		7.78 (1.26)		—		0.08 (–3.02 to 3.18)		.96	
**Patients with antiviral prescription and hospital admission on day 0 after DC visit (Tele-DC: n=9872; PDC: n=10,797)**											
	Overall admission rate, n (%)	20 (0.20)		20 (0.19)		0.01 (–0.10 to 0.13)		—		.78	
	LOS, mean (SE)	13.40 (4.84)		12.95 (3.70)		—		0.45 (–11.88 to 12.78)		.94	
**Patients without antiviral prescription and hospital admission on day 0 after DC visit (Tele-DC: n=7327; PDC: n=6402)**											
	Overall admission rate, n (%)	88 (1.20)		66 (1.03)		0.17 (–0.18 to 0.52)		—		.35	
	LOS, mean (SE)	13.84 (1.61)		9.82 (1.12)		—		4.02 (–0.11 to 8.16)		.06	
**Patients with antiviral prescription and AEDh visit (Tele-DC: n=9872; PDC: n=10,797), n (%)**											
	Within 1-28 days after DC visit	389 (3.94)		390 (3.61)		0.33 (–0.19 to 0.85)		—		.22	
	On day 0 after DC visit	35 (0.35)		48 (0.44)		–0.09 (–0.26 to 0.08)		—		.31	
**Patients without antiviral prescription and AED visit (Tele-DC: n=7327; PDC: n=6402), n (%)**											
	Within 1-28 days after DC visit	252 (3.44)		152 (2.37)		1.07 (0.51 to 1.63)		—		**<.001^i^**	
	On day 0 after DC visit	148 (2.02)		127 (1.98)		0.04 (–0.43 to 0.51)		—		.88	
**Patients with antiviral prescription and repeated DC visit (Tele-DC: n=9872; PDC: n=10,797), n (%)**											
	Second DC visit	906 (9.18)		856 (7.93)		1.25 (0.49 to 2.01)		—		**<.001^i^**	
	Third DC visit	96 (0.97)		95 (0.88)		0.09 (–0.17 to 0.35)		—		.49	
**Patients without antiviral prescription and repeated DC visit (Tele-DC: n=7327; PDC: n=6402), n (%)**											
	Second DC visit	540 (7.37)		431 (6.73)		0.64 (–0.22 to 1.50)		—		.15	
	Third DC visit	54 (0.74)		34 (0.53)		0.21 (–0.06 to 0.47)		—		.13	

^a^Tele-DC: tele-designated clinic.

^b^PDC: physical designated clinic.

^c^Between-group difference = Tele-DC group mean or percentage – PDC group mean or percentage.

^d^*P* values calculated with the chi-squared test.

^e^DC: designated clinic.

^f^Not applicable.

^g^LOS: length of stay.

^h^AED: accident and emergency department.

^i^Significant *P* values.

## Discussion

### Principal Findings

To the best of our knowledge, this is the first study to compare clinical outcomes between telemedicine and face-to-face consultations in managing patients with mild COVID-19 in a primary care setting using a large cluster-based cohort. Our findings revealed that the Tele-DC and PDC groups had comparable hospital admission rates, LOS, rates of severe complications, and mortality rates. However, the Tele-DC group exhibited a higher rate of visits at the AED and revisits at the DCs.

It is important to acknowledge a notable demographic difference between the Tele-DC and PDC groups prior to matching. The Tele-DC group consisted of younger individuals with fewer comorbidities, a lower prevalence of smoking, and a comparatively higher socioeconomic status. Similar findings have been observed in other studies comparing telemedicine groups with usual-care groups [26,27]. Telemedicine is a form of medical practice that heavily relies on internet technology as an extension of a doctor’s work environment [28]. Consequently, younger and more affluent individuals are more inclined to choose telemedicine as their preferred choice to receive medical care. To mitigate the impact of age, comorbidities, and socioeconomic status on clinical outcomes, we conducted matching between these two groups by age, sex, smoking status, CSSA status, and the Charlson comorbidity index. It should be noted that despite its potential, telemedicine can inadvertently widen health inequities by relying on digital access, a reliable internet connection, and digital literacy [29]. The use of telemedicine is limited by reduced access among socioeconomically disadvantaged populations who cannot afford the necessary devices or may not have internet access. Social justice and financial concerns that limit the equitable provision of services could be overcome through public funding and government investment [30].

Regarding our primary outcome, the comparable rates of hospital admission, LOS, and severe outcomes between the Tele-DC and PDC groups provide robust evidence that the lack of physical examination in a telemedicine setting does not compromise patient safety in mild COVID-19 cases. These findings suggest that for patients triaged as mild, clinical history taking and visual assessment via video or audio are sufficient to identify risk factors and manage the illness course. The similarity in HDU/ICU admissions and mortality rates further indicates that the Tele-DC model successfully identifies patients requiring escalation of care, with accuracy comparable to in-person clinicians, ensuring that high-risk patients are not missed despite the remote interface.

### Future Implications

The results of this study have significant implications for future pandemic preparedness and primary care resource allocation. First, establishing the clinical safety of Tele-DCs supports their use as a standard first line of defense during infectious disease surges, allowing health care systems to reserve physical clinic space and personal protective equipment for patients with more complex needs. Second, the data support a shift in policy toward a digital-first triage model for respiratory infections, reducing the risk of nosocomial transmission in waiting rooms. Finally, the comparable LOS suggests that patients managed remotely do not present to the hospital too late or in a worse condition than those seen in person, validating the effectiveness of the safety-net advice given during teleconsultations. Our findings align with the literature demonstrating the safety of telemedicine for acute respiratory infections. A study in Spain similarly reported that remote monitoring of patients with COVID-19 results in low hospitalization rates and low mortality rates, without an increase in adverse events compared to standard care [17].

However, as a physical examination could not be performed and some vital signs could not be checked for the Tele-DC group, the patients were understandably more inclined to seek face-to-face consultations at the AED for reassurance when their clinical condition changed. This may explain why the AED visit rate among patients in the Tele-DC group was slightly higher than that of the PDC group. This finding is similar to that of a previously large cohort study in the United States [31]. However, despite the higher AED visit rate, we did not observe a corresponding increase in the hospital admission rate. This suggests that most of the patients in the Tele-DC group visiting the AED did not exhibit severity warranting hospital admission.

The AED visit rate on day 0 after DC visits was similar between the two groups. The hospital admission rate on day 0 after Tele-DC visits was similar to that of the PDC group. This indicates that both groups have a similar ability to identify patients with potentially severe conditions requiring immediate AED or inpatient care. Although the PDC and Tele-DC were, indeed, designed to manage mild COVID-19, the disease’s dynamic nature means patients can rapidly deteriorate [32]. Our DC service serves as a critical safety net, identifying and escalating patients whose condition worsens. Therefore, potential severe cases with hospital admission or AED visits on the same day were not excluded but served as vital safety outcomes. These outcomes reflect the utility of the service in distinguishing patients suitable for community management and those requiring immediate higher-level care [33]. Excluding these cases would artificially inflate perceived success, introduce significant selection bias, and mask the real-world performance of our service. Their inclusion provides a more comprehensive and robust evaluation of our Tele-DC’s crucial role in patient management and safety.

The DC revisit rate for patients in the Tele-DC group after their initial visit was higher than that for the PDC group. This higher rate of follow-up visits may indicate that the patients’ needs were not adequately addressed at the first consultation, requiring additional unplanned care [15]. Beyond the lack of physical examinations, several other factors likely contributed to the increased AED visits and DC revisit rate in the Tele-DC group. First, provider caution and diagnostic uncertainty played a significant role. Faced with a novel and potentially rapidly progressing disease like COVID-19, and without the full spectrum of diagnostic information available in a face-to-face setting, doctors using telemedicine may have adopted a lower threshold for recommending an AED visit or an in-person follow-up. This cautious approach, although intended to ensure patient safety, can lead to overtriage and increased resource use. Second, patient anxiety and the need for reassurance cannot be underestimated. The psychological impact of a pandemic, coupled with the uncertainty surrounding COVID-19 symptoms, may have led patients to seek in-person care for reassurance [34], even for symptoms objectively deemed mild, if their virtual consultation did not fully alleviate their concerns. We did not have data on the exact reasons for AED visits and DC revisits.

These findings carry significant implications, particularly concerning the sufficiency of telemedicine for the triage of high-risk patients with COVID-19. Although our study demonstrated favorable outcomes for mild cases, the observed association with an increase in AED visits and DC revisits in the Tele-DC group strongly suggests its potential limitations for definitively triaging patients with moderate-to-severe symptoms or those at high risk of deterioration. The higher AED use, even if not accompanied by severe outcomes in our cohort, underscores a systemic challenge in balancing patient safety with efficient resource allocation when relying solely on virtual care for potentially complex presentations [12].

Interestingly, we observed 84.7% (1220/1446) patients continued with the Tele-DC after their initial visit, while only 71.0% (914/1287) of patients stuck to the PDC for their second visit. Several explanations may account for this observation. First, the Tele-DC provided easy access and saved travel time and money for patients. Therefore, if the purpose of the second visit was for symptomatic treatment of prolonged symptoms, patients were more likely to choose the Tele-DC. This was particularly true for individuals with limited mobility due to comorbidities, who were more prone to experiencing prolonged symptoms. Second, previous studies have demonstrated high patient satisfaction rates, shorter waiting times, and adequate communication with physicians in telemedicine settings [35,36]. As a result, ~80% of patients expressed willingness to choose telemedicine for future consultations after their initial visit [36].

However, the Tele-DC group exhibited a lower prescription rate of antivirals in both the overall patient population and older patients aged 60 years or above. According to the guideline for antiviral prescription from the Hong Kong Centre for Health Protection, antivirals are recommended for two groups: (1) patients ≥60 years old or <60 years old but with high-risk conditions, such as smoking, previous smoking history, or certain comorbidities, and (2) patients seen within 5 days since the onset of symptoms [37]. For Tele-DC consultations scheduled during the afternoon session, antivirals were delivered the next day. However, for patients in the PDC group, the antivirals were dispensed immediately after the consultation, regardless of whether it took place in the morning or in the afternoon. Based on the experience of Tele-DC doctors, a small proportion of patients in the Tele-DC group were not prescribed antivirals because their disease course would have exceeded 5 days by the next day of consultation. This may partly explain the lower prescription rate of antivirals observed in the Tele-DC group. Similarly, two previous studies focusing on telemedicine for managing acute upper respiratory tract infections, including sinusitis and pharyngotonsillitis, have found that the telemedicine leads to a lower prescription rate for antibiotics [26,38]. Therefore, Tele-DC doctors may have exercised caution in prescribing antivirals, considering the absence of a physical examination and a lack of vital sign assessment. Furthermore, the Tele-DC was popular and needed time to book. Maybe many patients in the Tele-DC group were already in days 3-4 of their disease course and, therefore, were not so eager to receive antiviral therapy. In addition, patients in the Tele-DC group were usually more well educated and health conscious; they were perhaps not willing to try the new antivirals due to their potential side effects, despite medical advice.

Our subgroup analysis revealed that both Tele-DC and PDC models were generally associated with comparable hospital admission rates, both on day 0 and within 1-28 days postvisits, regardless of whether patients received antiviral treatment. This suggests that Tele-DCs are associated with safety outcomes comparable to traditional face-to-face care for hospitalized patients with COVID-19 [39]. However, among patients without an antiviral prescription, the Tele-DC group exhibited a significantly higher rate of AED visits within 1-28 days. This finding suggests that although the Tele-DC may have been associated with similar hospital admission rates, it correlated with an increase in urgent care seeking in the absence of antiviral therapy, possibly due to a lack of direct physical assessment or differing thresholds for patient reassurance and escalation of care. Moreover, among patients receiving antiviral treatment, the Tele-DC was associated with a higher proportion requiring a second DC visit, potentially indicating a need for more frequent follow-up consultations to manage treatment or address ongoing concerns, even as admission rates and the LOS remained similar to those of the PDC.

Building on the discussion of potential health inequities, it is equally critical to acknowledge the profound ethical considerations inherent in telemedicine implementation. These include ensuring clinical safety in remote diagnostic and treatment scenarios, establishing robust data protection measures to safeguard sensitive patient information, developing clear and comprehensive protocols for informed consent in virtual care settings, maintaining and assessing professional competency for health care providers operating remotely, and upholding principles of distributive justice to ensure equitable access and quality of care for all populations [40]. A previous literature review found that the ethical and legal issues related to the practice of telehealth or telemedicine services still need standard and specific rules of application in order to guarantee equitable access, quality of care, sustainable costs, professional liability, respect of patient privacy, and data protection and confidentiality. In fact, telemedicine services could be used only as complementary or supplementary to the traditional health care services and not as a complete substitute [41].

### Strengths and Limitations

The main strength of this study is the inclusion of all eligible patients with COVID-19 at multiple public primary care clinics. This large sample size helped us ensure greater representativeness and reduced selection bias. Furthermore, we used a propensity score to match the two groups on factors that could potentially affect clinical outcomes, thereby minimizing confounding. Overall, these data analysis strategies contribute to the robustness of the findings and enhance the validity of the conclusions drawn.

This study also has several limitations. First, our participants were matched by age, smoking status, and existing comorbidities, which are known risk factors for severe COVID-19 [23]. Although various disease severity scores have been developed to predict outcomes in patients with COVID-19, such as the Quick COVID-19 Severity Index [42], the PRIEST COVID-19 Clinical Severity Score [43], the CHOSEN risk score for COVID-19 [44], and the 4C Mortality Score for COVID-19 [45], we did not use these severity scores to match our participants. This is because these scores were developed for hospitalized patients and were not applicable to our outpatient management approach. Our study lacks detailed, standardized data on well-established risk factors for severe COVID-19, such as the BMI; the duration, type, and severity of symptoms at initial presentation; and the duration of disease at presentation. Crucially, patients’ self-selected duration might have selectively chosen the PDC over the Tele-DC. As such, there may be residual confounding by indication that our matching process could not fully capture. Our study focused on mild patients, and those experiencing desaturation, respiratory distress, or symptoms of severe disease were advised to visit the AED for inpatient care. This unmeasured confounding remains a limitation.

Second, COVID-19 vaccination records were not included as baseline characteristics in this study due to data limitations. However, as of July 2022, ~94% of all Hong Kong residents had already received two doses of COVID-19 vaccines, with over 84% having received three doses [46]. Therefore, we have good reason to believe the vaccination rate between the two groups should be comparable. Third, a comprehensive socioeconomic status, including education levels and monthly incomes, was not included as a baseline variable in this study. These factors could be associated with smartphone usage rates and may have influenced the results. Fourth, there could be potential for misclassification bias arising from the use of ICPC-2 code A77 to identify COVID-19 cases. Because code A77 broadly encompasses other viral infections, such as Coxsackie virus; dengue fever; hand, foot, and mouth disease; and adenovirus, there is a possibility that patients without COVID-19 were inadvertently included in the cohort. Although we implemented strict mitigation measures, including restricting appointment bookings to self-reported confirmed COVID-19 cases and requiring nurses to verify the COVID-19 diagnostic method (PCR or RAT) during the initial assessment, the retrospective nature of the data means we cannot entirely rule out the chance of misclassification. Fifth, our study did not prospectively investigate the specific reasons for AED visits or DC revisits. Consequently, we were unable to definitively ascertain whether these visits were primarily driven by actual clinical deterioration, heightened patient anxiety, provider caution due to diagnostic uncertainty, or other factors (eg, non-COVID-19–related issues). This lack of detailed causal data limits our ability to distinguish between potentially avoidable visits and those that were clinically necessary, thereby obscuring clinically relevant distinctions regarding the precise drivers of utilization patterns.

Sixth, although doctors in both groups were drawn from the same pool of family physicians, the modality of consultation itself could have influenced clinical decision-making and treatment practices. The inherent information asymmetry in the Tele-DC, coupled with the heightened perceived risk and uncertainty during a pandemic, may have led to differences in antiviral prescription patterns, follow-up recommendations, or referral thresholds to emergency services. These unmeasured variations in physician practice, although not directly assessed in our study, could have subtly affected our observed outcomes, particularly the usage patterns. Seventh, we did not include a formal assessment of patient satisfaction with Tele-DC services. Although our findings shed light on clinical outcomes, understanding the patient experience is paramount for a comprehensive evaluation of telemedicine’s effectiveness and acceptability. Patient satisfaction directly influences adherence to treatment, engagement with care, and overall perception of health care quality, all of which are critical for the successful and sustainable integration of telemedicine into routine practice. Future research should prioritize the inclusion of validated patient satisfaction measures to provide a more holistic understanding of telemedicine’s impact from the end-user perspective, thereby informing strategies to optimize service delivery and improve patient engagement. Finally, our statistical approach used the chi-square test to compare binary outcomes over a fixed 28-day period. Although our centralized database ensured complete follow-up data for this window, this approach does not account for specific time-to-event dynamics or completing risks (eg, mortality) in the way that survival analyses such as Cox proportional hazards regression would. Future studies should incorporate time-to-event analyses to provide a more nuanced understanding of clinical trajectories.

### Conclusion

In conclusion, this study demonstrated that Tele-DCs were associated with clinical outcomes comparable to face-to-face consultations regarding hospitalization and severe complications among patients with mild COVID-19 in a primary care setting. Distinct from the existing literature that often focuses on complex remote patient monitoring using home devices, our work is innovative in validating a scalable, consultation-based model that relies primarily on clinical history rather than physical data. This brings to the field critical evidence that a physical examination is not strictly necessary for the safe triage of mild respiratory infections, challenging the traditional reliance on in-person assessment. The real-world implications of these findings support a digital-first strategy for future infectious surges to conserve resources and minimize transmission. However, the observed associations with higher AED visit and lower antiviral prescription rates highlight that telemedicine cannot operate in isolation; future implementation must integrate remote patient-monitoring tools and robust logistical support to fully replicate the efficacy of conventional care.

## Appendix

Multimedia Appendix 1 ICD-9-CM and ICPC-2 codes of comorbidities included in the Charlson comorbidity index and ICD-9-CM codes of severe complications of COVID-19. ICD-9-CM: *International Classification of Disease, Ninth Revision, Clinical Modification*; ICPC-2: International Classification of Primary Care-2.

## Abbreviations

AED: accident and emergency department
CDARS: Clinical Data Analysis and Reporting System
CMS: Clinical Management System
CSSA: comprehensive social security assistance
DC: designated clinic
ED: emergency department
GAI: generative artificial intelligence
GOPC: general outpatient clinic
HA: Hospital Authority
HDU: high-dependency unit
ICD-9-CM: *International Classification of Disease, Ninth Revision, Clinical Modification*
ICPC-2: International Classification of Primary Care-2
ICU: intensive care unit
LOS: length of stay
PCR: polymerase chain reaction
PDC: physical designated clinic
RAT: rapid antigen test
Tele-DC: tele-designated clinic

## Data Availability

The datasets generated and analyzed during the study are not publicly available as all the data are patients’ data under the Hong Kong Hospital Authority, but they are available from the corresponding author upon reasonable request.
