# Supplementary material for: Effectiveness of Telemedicine vs Face-to-Face Consultation in Fighting COVID-19: Retrospective Cohort Study of Adult Patients With COVID-19 in a Primary Care Setting
Source: J Med Internet Res. 2026 May 14;28:e74046. doi: 10.2196/74046 (PMC13219988; doi:10.2196/74046)
Supplement: Multimedia Appendix 1 [file jmir_v28i1e74046_app1.docx]

**Table S1.** ICD-9-CM, ICPC-2 codes of comorbidities included in Charlson comorbidity index.

| **Disease** | **ICPC-2** | **ICD-9-CM** |
| --- | --- | --- |
| Myocardial infarction | K75 | 410.x, 798.x |
| Congestive heart failure | K77 | 428.x |
| Peripheral vascular disease | K92 | 250.6x, 997.2, 997.6x, 440.2x |
| Stroke or transient ischemic attack | K89-K91 | 430.x-438.x |
| Dementia | P70 | 290.0-290.9 |
| Chronic Obstructive Pulmonary Disease | R79, R95 | 491.x, 492.x, 493.2x ,496 |
| Connective tissue disease | L88, L99 | 710 |
| Peptic ulcer disease | D85, D86 | 531.0-534.91 |
| Hepatitis | D72 | 573.1-573.3, 571.4 |
| Cirrhosis | D97 | 571.2, 571.5, 571.6 |
| Diabetes mellitus | T89, T90 | 250 |
| Hemiplegia | N99 | 342.00-342.92 |
| ESRD | NA | 250.3x, 585.x, 586 |
| Solid tumor | D77,R85,D74,D75,D76, R85,H75,R84, B72, B73, B74, K72,A79,R85,L71, S77,N74,X76,Y78,X77, X75,W72, Y78,U75,U77, U76,F74,T71,T73 | 140-209 |
| Leukemia | B73 | 204.00-208.92 |
| Lymphoma | B72 | 201-202 |
| Acquired immunodeficiency Syndrome | B90 | 42 |
| Asthma | R96 | 493 |

**Table S2.** ICD-9-CM codes of severe complications of COVID-19.

| **Disease** | **ICD-9-CM** |
| --- | --- |
| Pneumonia | 480-486 |
| Sepsis | 995.91 |
| Encephalopathy | 348.3 |
| Encephalitis | 323 |
| Myocarditis | 422 |
| Systemic inflammatory response syndrome | 995.9 |
| Shock | 785.5 |
